# Regioselectivity in the Ring Opening of Epoxides for the Synthesis of Aminocyclitols from D-(−)-Quinic Acid

**DOI:** 10.3390/molecules17044498

**Published:** 2012-04-16

**Authors:** Tzenge-Lien Shih, Shu-Yu Yang

**Affiliations:** Department of Chemistry, Tamkang University, No151 Yingzhuan Rd, 25137 Tamsui Dist., New Taipei City, Taiwan; Email: 699180088@s99.tku.edu.tw

**Keywords:** aminocyclitols, epoxides, glycosidase inhibitors, D-(−)-quinic acid, regioselective ring opening

## Abstract

Efficient syntheses of four aminocyclitols are reported. Each synthesis is accomplished in eight steps starting from D-(−)-quinic acid. The key step involves a highly regioselective ring opening of epoxides by sodium azide.

## 1. Introduction

**Figure 1:**
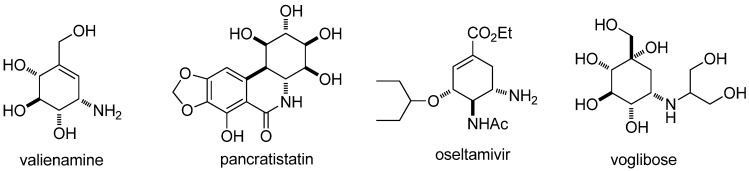
Representative natural or synthetic products containing aminocyclitol moiety.

Aminocyclitols, also known as aminocarbasugars [1], contain at least one amino or substituted amino moiety in the cyclitols (polyhydroxylated cycloalkanes) [2]. Many natural and synthetic products containing aminocyclitol scaffolds have shown a variety of biological activities [3,4], such as, valienamine [5], pancratistatin [6], oseltamivir [7], and voglibose [4] (Figure 1). The synthesis of biological active aminocyclitols and assessment of their structure and activity relationship have generated considerable interest in recent years [4,8,9,10,11,12,13,14,15,16,17].

**Figure 2:**
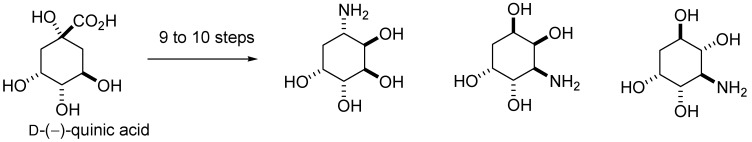
The previously synthesized aminocyclitols.

Previously, we have synthesized three aminocyclitols from D-(−)-quinic acid in nine to ten steps via stereoselective dihydroxylation as a key step [18] (Figure 2). These quercitol-like structures of aminocyclitols are also called as deoxyinosamines [4]. We described herein an alternative synthesis of two known aminocyclitols **5** and **6** along with two new aminocyclitols **10** and **11**. The synthesis was accomplished in eight steps via a regioselective ring opening reaction of epoxides.

## 2. Results and Discussion

**Scheme 1:**
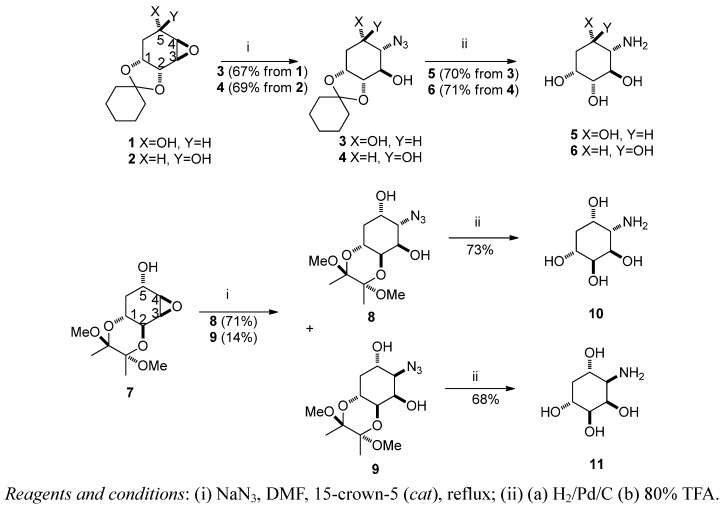
Synthesis of aminocyclitols **5**, **6**, **10** and **11**.

Unlike the strategy we previously used in the synthesis of aminocyclitols (Figure 2), we started from the epoxides **1**, **2** and **7**, which were prepared from D-(−)-quinic acid in six steps, respectively [19]. When compounds **1** and **2** were treated with sodium azide in DMF under reflux conditions, they underwent a highly regioselective opening at the C4 position to afford **3** and **4**, respectively (Scheme 1).

**Figure 3:**
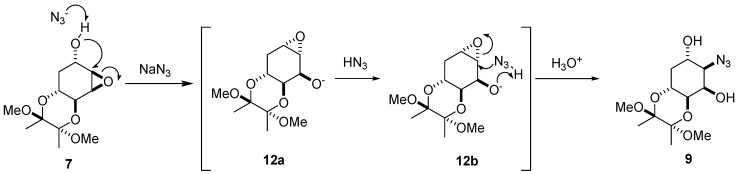
Plausible mechanism for the formation of **9**.

The yields were mediocre but no other regioisomers were detected by TLC or isolated from column purification [20]. Interestingly, the TMB-protected compound **7** was treated with NaN_3_ to afford **8** in 71% yield and its epimer **9** in 14% yield. The azide directly attacked the least hindered side of **7** at the C4 position to give **8**. However, a plausible mechanism for the formation of the minor component **9** results from the C5 hydroxide group of **7** being attacked at the C4 position to give intermediate **12a** (Figure 3).

**Figure 4:**
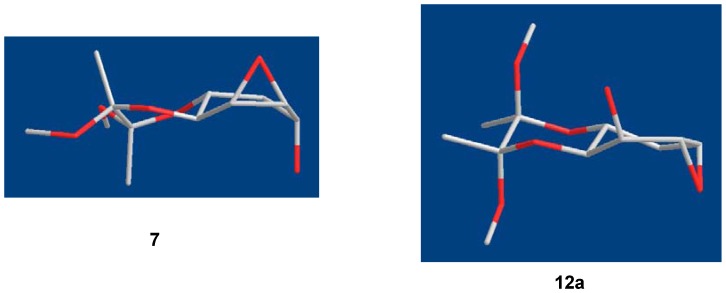
Three-dimensional representations of structures **7** and **12a**.

Instead of attack at the least hindered side at the C5 position of **12a** by azide, known as the Payne rearrangement [21], the hydroxide group at C3 of **12a** internally removed the proton of HN_3_ (intermediate **12b**). That allowed the resulting azide to attack the vicinal C4 position of **12b** to give **9**. This resulted in the retention of configuration of epoxide **7**. This observation was very unusual and in contrast to the results that occurred in the 2,3-epoxy rearrangement [22]. Based on the Chem3D simulation, the cyclohexane core of **7** was in a boat-like conformation (Figure 4). The trans-diaxial attack at C4 in **7** by azide leading to **8** as the major compound was energetically favorable. However, we could not rule out the possibility in formation of **12a** which was derived from the trans-axial attack of the epoxide by C5-OH in **7**. The lower yield of **9** was probably due to the half-chair like structure **12a** that was less favorable than **7** for allowing by azide attack (Figure 4).

**Figure 5:**
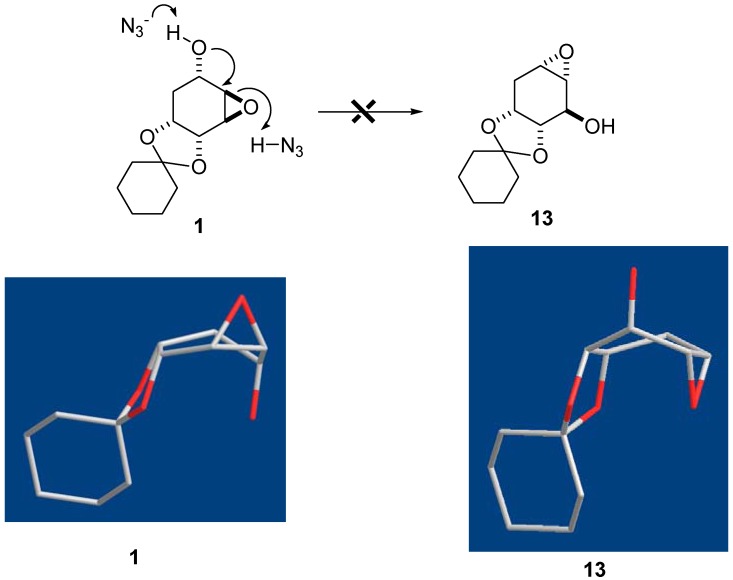
Three-dimensional representations of structures **1** and **13**.

The Payne rearrangement of epoxide **7** intrigued us as an interesting issue when no rearrangement product **13** was found when compound **1** was treated with NaN_3_ (Figure 5). According to the Chem3D simulation, the conformation of cyclohexane core of **1** is a slightly twisted boat form. However, compound **13** was in a boat conformation if the Payne rearrangement occurred. The reason was probably due to the steric congestion in the formation of **13** because the distance between epoxide and the C2 acetal oxygen atom of **13** is around 3.054 Å. On the contrary, the distance between the C5-OH and C2 oxygen atom of **1** is about 3.328 Å. Therefore, the trans-diaxial attack at C4 of **1** by azide might be kinetically or sterically controlled to lead to the major component **3**.

In order to obtain better yields of final products **5**, **6**, **10**, and **11**, we determined that azido compounds **3**, **4**, **8**, and **9** should be hydrogenated first over Pd/C, followed by deprotection under acidic conditions. The one pot reaction conditions (H_2_/Pd/C/HCl) afforded low yields of target compounds accompanied by a more complicated mixture. It is worth noting that our strategy was much shorter than the reported method in the syntheses of molecules **5** and **6** which involved sixteen steps starting from D-mannitol [23]. The structure determinations were based on a series of NMR experiments (COSY, 2D-NOESY, HMBC, HMQC, and HRMS) and the selected NMR data were listed on Table 1 and Table 2.

**Table 1:** Selected ^1^H (600 MHz) and ^13^C (150 MHz) NMR data for **3**, **4**, **8**, and **9** in CD_3_OD. 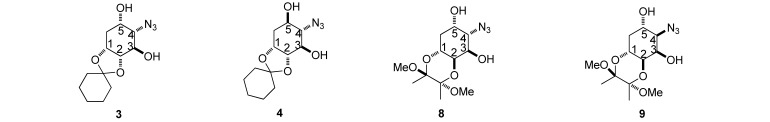

Compound	H_1_ ( *J*)/C_1_	H_2_ ( *J*)/C_2_	H_3_ ( *J*)/C_3_	H_4_ ( *J*)/C_4_	H_5_ ( *J*)/C_5_	H_6_ ( *J*)/C_6_
**3**	4.33–4.30 (m) 74.7	3.92 (dd, 7.0, 5.3) 81.4	4.04–4.01 (m) 69.3	3.21 (dd, 10.0, 2.7) 86.8	4.07–4.04 (m) 71.9	2.25 (dt, 15.7, 3.6) 1.93 (ddd, 15.7, 7.1, 3.7) 32.1
**4**	4.30 (dt, 6.5, 2.3) 74.1	3.89 (dd, 7.6, 5.2) 81.0	3.38 (dd, 10.4, 7.6) 76.1	3.04 (t, 10.2) 71.5	3.62 (td, 11.2, 4.9) 68.6	2.34 (ddd, 14.9, 4.9, 2.2) 1.75 (ddd, 14.9, 11.5, 4.1) 34.1
**8**	3.88 (ddd, 11.6, 10.1, 4.7) 65.3	3.95 (dd, 10.0, 3.4) 71.1	3.85 (t, 3.5) 64.5	3.79 (t, 3.0) 72.5	3.74 (ddd, 11.6, 5.2, 3.0) 67.6	1.79–1.74 (m) 1.71 (t, 11.6) 32.8
**9**	3.51–3.40 (m) 66.1	3.78 (tm, 9.5, 0.8) 75.0	3.32 (td, 9.4, 0.8) 72.3	3.13 (td, 9.5, 1.7) 73.0	3.45–3.42 (m) 69.7	2.01 (dt, 12.1, 4.6) 1.51 (ddd, 13.3, 12.3, 1.4) 36.3

**Table 2:** Selected ^1^H (600 MHz) and ^13^C (150 MHz) NMR data for **5**, **6**, **10**, and **11** in D_2_O. 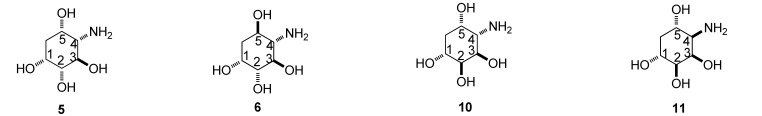

Compound	H_1_ ( *J*)/C_1_	H_2_ ( *J*)/C_2_	H_3_ ( *J*)/C_3_	H_4_ ( *J*)/C_4_	H_5_ ( *J*)/C_5_	H_6_ ( *J*)/C_6_
**5**	4.03 (dd, 6.3, 3.1) 70.0	3.45 (dd, 9.4, 2.8) 73.8	3.90 (t, 9.8) 67.2	3.12 (d, 10.3, 3.2) 56.7	4.09 (dd, 6.5, 3.2) 66.9	2.08 (dt, 15.6, 3.5) 1.70 (dt, 15.6, 2.9) 32.6
**6**	3.96–3.94 (m) 68.3	3.41–3.34 (m)^a^ 73.9	3.41–3.34 (m)^a^ 71.7	2.53 (t, 9.8) 59.4	3.59 (ddd, 14.5, 10.0, 4.6) 67.6	1.98 (dt, 14.0, 4.2) 1.47 (td, 14.0, 2.5) 36.3
**10**	3.91 (dt, 9.1, 3.5) 67.0	3.75–3.65 (m)^a^ 68.3	3.75–3.65 (m)^a^ 67.2	3.12 (t, 4.0) 52.6	3.75–3.65 (m)^a^ 72.1	1.85 (td, 13.1, 4.1) 1.75–1.62 (m) 32.9
**11**	3.39 (ddd, 11.9, 9.4, 4.6) 68.6	3.15 (t, 9.3) 77.0	2.99 (t, 9.6) 74.0	2.49 (d, 9.8) 58.8	3.30 (td, 11.4, 4.4) 68.7	2.06 (dt, 12.2, 4.5) 1.35 (dd, 11.9, 11.9) 68.6

^a^ Assignments were not well resolved due to signal overlaps.

## 3. Experimental

### 3.1. General Methods

^1^H (600 MHz) and ^13^C-NMR (150 MHz) spectra were recorded on a Bruker 600 MHz instrument. The chemical shifts were reported in ppm and relative to the residual of *d*-solvents: CD_3_OD (^1^H, 4.78 ppm; ^13^C, 49.0 ppm); D_2_O (4.69 ppm). Optical rotations were measured with a HORIBA SEPA-300 instrument. HRMS were measured by a Finnigan MAT 95S spectrometer.

### 3.2. General Procedure of Ring Opening

Compound **1** (0.838 g, 4.0 mmol), for example, was dissolved in DMF (30 mL). To this mixture was added NaN_3_ (2.3 g, 36.0 mmol) and a catalytic amount of 15-crown-5 and heated under reflux for 5−6 h. At the end of the reaction time, the mixture was diluted with H_2_O (100 mL) and extracted with Et_2_O (2x100 mL). The organic layer was dried (MgSO_4_) and purified by column chromatography.

### 3.3. General Procedures of Hydrogenation and Deprotection

Compound **3** (0.079 g, 0.29 mmol) for example, was dissolved in MeOH (2 mL). To this mixture was added 10% Pd/C (10 mol%) and it was hydrogenated under one atmosphere at ambient temperature for 2 h. The resulting mixture was filtrated through a pad of Celite and washed with MeOH. The organic layer was concentrated and 80% TFA was added (2 mL), then stirred for 1−1.5 h, at the end of which time, the solvent was evaporated and the residue purified by column chromatography.

### 3.4. Synthesis of the Key Intermediates and the Target Molecules

#### 3.4.1. (1R,2R,3R,4S,5S)-4-Azido-1,2-O-cyclohexylidene-cyclohexane-1,2,3,5-tetraol (**3**)

Purification by flash column chromatography (230–400 mesh SiO_2_, EtOAc/hex = 1/8−1/2) afforded a white solid. Yield = 67%. MP = 122−128 °C. [*α*]_D_^25^ +36.2 (*c* 0.31, MeOH). ^1^H-NMR (CD_3_OD)*δ *4.56 (s, 2H, -OH), 4.33−4.30 (m, 1H), 4.07−4.04 (m, 1H), 4.04−4.01 (m, 1H), 3.92 (dd, *J* = 7.0, 5.3 Hz, 1H), 3.21 (dd, *J* = 10.0, 2.7 Hz, 1H), 2.25 (dt, *J* = 15.7, 3.6 Hz, 1H), 1.93 (ddd, *J* = 15.7, 7.1, 3.7 Hz, 1H), 1.72−1.54 (m, 8H), 1.48−1.42 (m, 2H). ^13^C-NMR (CDCl_3_) *δ *110.7, 81.4, 74.7, 71.9, 69.3, 66.8, 39.4, 36.2, 32.1, 26.2, 25.1, 24.8. HRMS (ESI) calcd for C_12_H_19_N_3_O_4_ (M^+^) 269.1376. Found: 269.1371.

#### 3.4.2. (1R,2R,3R,4S,5R)-4-Azido-1,2-O-cyclohexylidene-cyclohexane-1,2,3,5-tetraol (**4**)

Purification by flash column chromatography (230−400 mesh SiO_2_, EtOAc/hex = 1/8−1/2) afforded a white solid. Yield = 69%. Mp = 125−130 °C. [*α*]_D_^25^−146.6 (*c *0.45, MeOH). ^1^H-NMR (CD_3_OD) *δ *4.57 (s, 2H, -OH), 4.30 (dt, *J* = 6.5, 2.3 Hz,1H), 3.89 (dd, *J* = 7.6, 5.2 Hz, 1H), 3.62 (td, *J* = 11.2, 4.9 Hz, 1H), 3.38 (dd, *J* = 10.4, 7.6 Hz, 1H), 3.04 (t, *J* = 10.2 Hz, 1H), 2.34 (ddd, *J* = 14.9, 4.9, 2.2 Hz, 1H), 1.75 (ddd, *J* = 14.9, 11.5, 4.1 Hz, 1H), 1.70−1.52 (m, 8H), 1.44−1.40 (m, 1H), 1.39−1.30 (m, 1H).^13^C-NMR (CD_3_OD) *δ *110.8, 81.0, 76.1, 74.1, 71.5, 68.6, 39.3, 36.2, 34.1, 26.1, 25.0, 24.8. HRMS (ESI) calcd for C_12_H_19_N_3_O_4_ (M^+^) 269.1376. Found: 269.1377.

#### 3.4.3. (1R,2S,3R,4S,5S)-4-Azido-1,2-[(2S,3S)-2,3-dimethoxybutan-2,3-dioxy]-cyclohexane-1,2,3,5-tetraol (**8**)

Purification by flash column chromatography (230–400 mesh SiO_2_, EtOAc/hex = 1/15−1/2) afforded a white solid. Yield = 71%. MP = 178−182 °C. [*α*]_D_^25^ +157.4 (*c* 0.19, MeOH). ^1^H-NMR (CD_3_OD) *δ *3.95 (dd, *J* = 10.0, 3.4 Hz, 1H), 3.88 (ddd, *J* = 11.6, 10.1, 4.7 Hz, 1H), 3.85 (t, *J* = 3.5 Hz, 1H), 3.79 (t, *J* = 3.0 Hz, 1H), 3.74 (ddd, *J* = 11.6, 5.2, 3.0 Hz, 1H), 3.24 (s, 6H), 1.79−1.74 (m, 1H), 1.71 (t, *J* = 11.6 Hz, 1H), 1.28 (s, 3H), 1.24 (s, 3H). ^13^C-NMR (CD_3_OD) *δ *101.4, 100.6, 72.5, 71.1, 67.6, 65.3, 64.5, 48.2, 48.1, 32.8, 18.0, 17.9. HRMS (ESI) calcd for C_12_H_21_N_3_NaO_6_ [M+Na]^+^ 326.1328. Found: 326.1308.

#### 3.4.4. (1R,2S,3R,4R,5S)-4-Azido-1,2-[(2S,3S)-2,3-dimethoxybutan-2,3-dioxy]-cyclohexane-1,2,3,5-tetraol (**9**)

Purification by flash column chromatography (230–400 mesh SiO_2_, EtOAc/hex = 1/15−1/2) afforded a white solid. Mp = 179−185 °C. Yield = 14%. [*α*]_D_^25^ +164.3 (*c* 0.28, MeOH). ^1^H-NMR (CD_3_OD) *δ *3.78 (td, *J* = 9.5, 0.8 Hz, 1H), 3.51−3.40 (m, 2H), 3.32 (td, *J* = 9.4, 0.8 Hz, 1H), 3.27 (s, 3H), 3.21 (s, 3H), 3.13 (td, *J* = 9.5, 1.7 Hz, 1H), 2.01 (dt, *J* = 12.1, 4.6 Hz, 1H), 1.51 (ddd, *J* = 13.3, 12.3, 1.4 Hz, 1H), 1.27 (s, 3H), 1.24 (s, 3H). ^13^C-NMR (CD_3_OD) *δ *100.7 (×2), 75.0, 73.2, 72.3, 69.7, 66.1, 48.3, 48.2, 36.3, 17.9 (x2). HRMS (ESI) calcd for C_12_H_21_N_3_NaO_6_ [M+Na]^+^ 326.1328. Found: 326.1328.

#### 3.4.5. (1R,2R,3R,4S,5S)-4-Aminocyclohexane-1,2,3,5-tetraol (**5**)

Purification by flash column chromatography (230–400 mesh SiO_2_, MeOH/CH_2_Cl_2_/5%NH_4_OH = 1/10−1/1) afforded a pale yellow syrup. Yield = 70%. [*α*]_D_^25^−76.7 (*c* 0.21, H_2_O). ^1^H-NMR (D_2_O) *δ *4.09 (dd, *J* = 6.5, 3.2 Hz, 1H), 4.03 (dd, *J* = 6.3, 3.1 Hz, 1H), 3.90 (t, *J* = 9.8 Hz, 1H), 3.45 (dd, *J* = 9.4, 2.8 Hz, 1H), 3.12 (dd, *J* = 10.3, 3.2 Hz, 1H), 2.08 (dt, *J* = 15.6, 3.5 Hz, 1H), 1.70 (dt, *J* = 15.6, 2.9 Hz, 1H). ^13^C-NMR (D_2_O) *δ* 73.8, 70.0, 67.2, 66.9, 56.7, 32.6. HRMS (ESI) calcd for C_6_H_14_NO_4_ [M+H]^+^ 164.0923. Found: 164.0919.

#### 3.4.6. (1R,2R,3R,4S,5R)-4-Aminocyclohexane-1,2,3,5-tetraol (**6**)

Purification by flash column chromatography (230–400 mesh SiO_2_, MeOH/CH_2_Cl_2_/5%NH_4_OH = 1/10−1/1) afforded a pale yellow syrup. Yield = 71%. [*α*]_D_^25^−19.4 (*c* 0.33, H_2_O). ^1^H-NMR (D_2_O) *δ *3.96−3.94 (m, 1H), 3.59 (ddd, *J* = 14.5, 10.0, 4.6 Hz, 1H), 3.41−3.34 (m, 2H), 2.53 (t, *J* = 9.8 Hz, 1H), 1.98 (dt, *J* = 14.0, 4.2 Hz, 1H), 1.47 (td, *J* = 14.0, 2.5 Hz, 1H). ^13^C-NMR (D_2_O) *δ *73.9, 71.7, 68.3, 67.6, 59.4, 36.3. HRMS (ESI) calcd for C_6_H_13_NO_4_ (M^+^) 163.0845. Found: 163.0835.

#### 3.4.7. (1R,2S,3R,4S,5S)-4-Aminocyclohexane-1,2,3,5-tetraol (**10**)

Purification by flash column chromatography (230–400 mesh SiO_2_, MeOH/CH_2_Cl_2_/5%NH_4_OH = 1/10−1/1) afforded a pale yellow syrup. Yield = 73%. [*α*]_D_^25^−48.2 (*c* 0.19, H_2_O). ^1^H-NMR (D_2_O) *δ* 3.91 (dt, *J *= 9.1, 3.5 Hz, 1H), 3.75−3.65 (m, 3H), 3.12 (t, *J* = 4.0 Hz, 1H), 1.85 (dt, *J* = 13.1, 4.1 Hz, 1H), 1.75−1.62 (m, 1H). ^13^C-NMR (D_2_O) *δ *72.1, 68.3, 67.2, 67.0, 52.6, 32.9. HRMS (ESI) calcd for C_6_H_14_NO_4_ [M+H]^+^ 164.0923. Found: 164.0920.

#### 3.4.8. (1R,2S,3R,4R,5S)-4-Aminocyclohexane-1,2,3,5-tetraol (**11**)

Purification by flash column chromatography (230–400 mesh SiO_2_, MeOH/CH_2_Cl_2_/5%NH_4_OH = 1/10−1/1) afforded a pale yellow syrup. Yield = 68%. [*α*]_D_^25^−69.4 (*c* 0.18, H_2_O). ^1^H-NMR (D_2_O) *δ *3.39 (ddd, *J *= 11.9, 9.4, 4.6 Hz, 1H), 3.30 (td, *J *= 11.4, 4.4 Hz, 1H), 3.15 (t, *J* = 9.3 Hz, 1H), 2.99 (t, *J *= 9.6 Hz, 1H), 2.49 (t, *J *= 9.8 Hz, 1H), 2.06 (dt, *J* = 12.2, 4.5 Hz, 1H), 1.35 (dd, *J* = 11.9, 11.9 Hz, 1H). ^13^C-NMR (D_2_O) *δ *77.3, 74.0, 68.7, 68.6, 58.8, 38.0. HRMS (ESI) calcd for C_6_H_14_NO_4_ [M+H]^+^ 164.0923. Found: 164.0918.

## 4. Conclusions

In conclusion, aminocyclitols are a very important class of aminocarbasugars. We have synthesized two known and two new aminocyclitols in an efficient manner from D-(−)-quinic acid. Especially, our method provided a short alternative in syntheses of **5** and **6** than the literature. The ring opening of epoxide in **1**, **2** and **7** by sodium azide to provide moderate to good yields of **3**, **4**, and **8**, respectively, was highly regioselective owing to the steric effect. The studies of the biological activities of these compounds are currently ongoing and will be reported in due course.
